# Crystal Structure of a Thermostable Alanine Racemase from *Thermoanaerobacter tengcongensis* MB4 Reveals the Role of Gln360 in Substrate Selection

**DOI:** 10.1371/journal.pone.0133516

**Published:** 2015-07-28

**Authors:** Xiaoliang Sun, Guangzheng He, Xiaoyan Wang, Shujing Xu, Jiansong Ju, Xiaoling Xu

**Affiliations:** 1 Institute of Ageing Research, School of Medicine, Hangzhou Normal University, Hangzhou, China; 2 College of Life Science, Hebei Normal University, Shijiazhuang, China; University of Cantebury, NEW ZEALAND

## Abstract

Pyridoxal 5’-phosphate (PLP) dependent alanine racemase catalyzes racemization of L-Ala to D-Ala, a key component of the peptidoglycan network in bacterial cell wall. It has been extensively studied as an important antimicrobial drug target due to its restriction in eukaryotes. However, many marketed alanine racemase inhibitors also act on eukaryotic PLP-dependent enzymes and cause side effects. A thermostable alanine racemase (Alr_Tt_) from *Thermoanaerobacter tengcongensis* MB4 contains an evolutionarily non-conserved residue Gln360 in inner layer of the substrate entryway, which is supposed to be a key determinant in substrate specificity. Here we determined the crystal structure of Alr_Tt_ in complex with L-Ala at 2.7 Å resolution, and investigated the role of Gln360 by saturation mutagenesis and kinetic analysis. Compared to typical bacterial alanine racemase, presence of Gln360 and conformational changes of active site residues disrupted the hydrogen bonding interactions necessary for proper PLP immobilization, and decreased both the substrate affinity and turnover number of Alr_Tt_. However, it could be complemented by introduction of hydrophobic amino acids at Gln360, through steric blocking and interactions with a hydrophobic patch near active site pocket. These observations explained the low racemase activity of Alr_Tt_, revealed the essential role of Gln360 in substrate selection, and its preference for hydrophobic amino acids especially Tyr in bacterial alanine racemization. Our work will contribute new insights into the alanine racemization mechanism for antimicrobial drug development.

## Introduction

Alanine racemase (Alr, EC 5.1.1.1) is a fold-type III pyridoxal 5’-phosphate (PLP)-dependent enzyme that catalyzes the reversible racemization of L-Ala and D-Ala. D-Ala is one of the key building blocks of peptidoglycan network, an elastic polymer layer consisting of sugars and amino acids that forms the bacteria cell wall [1], protecting cell against osmotic pressure and attack from L-amino acids targeted peptidases [1, 2]. Due to its absence in most higher eukaryotes, alanine racemase has long been considered as an important antimicrobial drug target [3–7]. However, many alanine racemase inhibitors covalently bind to some eukaryotic PLP-dependent enzymes [8–12], and cause side effects [13]. Thus, understanding the racemization mechanism of alanine racemase, especially the determinant of substrate specificity, remains an urgent need for antimicrobial drug development.

In bacteria, two types of alanine racemase are encoded independently by two genes named *dadX* and *alr* [14, 15]. The *dadX* gene encodes a catabolic alanine racemase DadX, which catalyzes direct conversion of L-Ala to D-Ala. Its expression is induced by L- or D-Ala [16, 17]. However, the *alr* gene encodes an anabolic alanine racemase Alr, it is expressed constitutively at low level and essential for providing abundant D-Ala for peptidoglycan biosynthesis [14]. *Mycobacterium smegmatis* strains with a deletion of the *alr* gene require D-Ala for growth, indicating the essential role of Alr in D-Ala production [18]. It is reported that the catabolic alanine racemase DadX usually shows much higher catalytic efficiency than the anabolic enzyme Alr [19, 20]. Some bacteria only contain one type alanine racemase gene, whereas others have two of them. However, the reason of having one or two alanine racemase genes in one organism is still not fully understood.

*Thermoanaerobacter tengcongensis (T*. *tengcongensis)* MB4 is an anaerobic gram-negative bacterium isolated from a hot spring in Tengchong, Yunnan province of China [21]. Its optimal propagation occurs at 75°C, with pH values ranging from 7 to 7.5 [21]. *T*. *tengcongensis* MB4 contains two annotated alanine racemase genes *MBalr1* (AAM24437.1) and *MBalr2* (AAM25327.1). Both genes encode 388 amino acids long alanine racemase, sharing a 58.3% amino acid sequence identity [22]. Compared with MBAlr2, MBAlr1 shows very low catalytic efficiency and limited substrate spectrum [22]. It is probable that MBAlr1 serves as an anabolic and MBAlr2 as the catabolic alanine racemase in *T*. *tengcongensis* MB4 [22]. For clarity, we name MBAlr1 as Alr_*Tt*_ and MBAlr2 as DadX_*Tt*_ in this work.

To date, crystal structures of alanine racemases and their complexes with substrates and analogs from several microorganisms have been reported [12, 23–33]. The substrate entryway to the active site of alanine racemase is highly conserved across species, it could be divided into three layers (outer, middle and inner) [24]. Based on multiple sequence alignment and structure analysis, the inner and middle layers of most alanine racemase are comprised of 8 strongly conserved residues [24, 31, 34]. In Alr_*Tt*_, the middle (Arg293’, Arg313’ and Ile358’) and inner layer (Tyr287’, Tyr268’ and Ala172) residues are strictly conserved among bacterial alanine racemase, except two residues (Ser173 in middle layer and Gln360 in inner layer). Especially, Gln360 is the only inner layer residue that is not conserved in both Alr_*Tt*_ and DadX_*Tt*_ (Alr_*Tt*_: Gln360; DadX_*Tt*_: His359), which is replaced by Tyr in other bacterial alanine racemase. It has been reported that Tyr354 of *Geobacillus stearothermophilus* alanine racemase is essential in determining the substrate specificity [35]. In *T*. *tengcongensis* MB4, mutation at Gln360 (Gln360→Tyr360) of Alr_*Tt*_ resulted in a 147.9% increase of the enzyme activity [36]. However, mutation of His359 (His359→Tyr359) dramatically decreased the racemase activity of DadX_*Tt*_ [22]. These observations draw our attention to the role of Gln360 in alanine racemization.

In order to understand the biochemical properties of Alr_*Tt*_, especially the role of the non-conserved residue Gln360 in alanine racemization, we determined the crystal structure of Alr_*Tt*_ in complex with L-Ala at 2.7 Å resolution, and investigated its racemase activity by saturation mutagenesis of Gln360. Overall architecture of Alr_*Tt*_ is similar to typical bacterial alanine racemase. However, presence of Gln360 and conformational changes of the active site residues destabilized PLP immobilization, resulted in the low racemase activity of Alr_*Tt*_. Introduction of hydrophobic amino acids at Gln360 increased the overall catalytic efficiency (*k*_cat_/*K*_m_) of the enzyme. Through interactions with a hydrophobic patch near the active site pocket, these hydrophobic amino acids, especially Tyr residue steric block the entry and turnover of larger amino acids, enhance the substrate specificity of Alr_*Tt*_. This work for the first time revealed a preference of hydrophobic amino acids at Gln360 position for substrate selection, further explored the racemization mechanism of bacterial alanine racemase, it will contribute useful information for antibiotics development.

## Materials and Methods

### Construction of the expression plasmids for wild-type and mutant Alr_*Tt*_

The alanine racemase gene *alr*_*Tt*_ (GenBank: AAM24437.1) was amplified by PCR using the genomic DNA of *T*. *tengcongensis* MB4 as template and a pair of primers (S1 Table: Alr_*Tt*_-F’ and Alr_*Tt*_-R’). PCR products were purified using gel extraction kit (Tiangen, China), and then inserted into TA cloning vector pMD19-T to construct plasmid pMD-Alr_*Tt*_. The plasmid was verified by DNA sequencing and digested with restriction enzyme *Nhe*I and *Xho*I, the gene fragment encoding full-length Alr_*Tt*_ was then ligated into a pET-28a vector (Novagen) to obtain the expression plasmid pET-28a-Alr_*Tt*_.

The QuikChange Site-directed Mutagenesis kit (Stratagene, USA) was used for mutation of residues Ser173 to Asp173 (TCC→GAC), Gln360 to Tyr360 (CAA→TAT) and Gln360 to other residues (saturation mutagenesis, CAA→NNS) in the substrate entryway of Alr_*Tt*_. PCR products were obtained with primer pairs S173D-F’ and S173D-R’, Q360Y-F’ and Q360Y-R’, S-Q360-F’ and S-Q360-R’ summarized in S1 Table.

Nucleotide sequences of the expression plasmids of wild-type and mutant Alr_*Tt*_ were determined using an ABI 3730xl DNA sequencer (Applied Biosystems). Constructs containing correct gene sequences were transformed into *E*.*coli* BL21(DE3) for enzyme expression, purification and racemization assay.

### Enzyme expression and purification

Full length Alr_*Tt*_ was cloned into pET28a vector with both N- and C-terminal 6×His tag and overexpressed in *E*. *coli* BL21 (DE3) cells. The cells were lysed by sonication in buffer containing 50 mM NaH_2_PO_4_ pH8.0, 300 mM NaCl and 20 mM imidazole. The cell lysates were centrifuged at 26,664 g for 10 min. The soluble fractions were applied to Ni-NTA column (GE healthcare), and the bound Alr_*Tt*_ was eluted by 250 mM imidazole. After buffer exchange into 25 mM Tris-HCl pH8.0, 200 mM NaCl, 10 μM PLP, Alr_*Tt*_ was further purified by size exclusion chromatography using a Superdex 200 10/300 GL column (GE Healthcare). The purified Alr_*Tt*_ was collected and dialyzed against 10 mM Tris-Cl pH8.0, 10 μM PLP, and concentrated with an Amicon Ultra-15 Centrifugal Filter Device (Millipore) for crystallization. Enzyme expression and purification of Alr_*Tt*_ mutants were same as wild-type.

### Crystallization

Crystallization was performed using hanging-drop vapor diffusion method. Purified Alr_*Tt*_ (10 mg mL^-1^) was incubated with L-Ala and PLP at 1:1.5:1.5 molar ratio at 277 K for 2 h before crystallization. Diffracted crystals were obtained at 289 K by mixing 1 μL L-Ala and PLP incubated Alr_*Tt*_ with 1 μL reservoir solution (22% PEG 4000, 0.1 M Bis-Tris pH7.0) and equilibrating the mixture against 300 μL reservoir solution.

### Diffraction data collection, structure determination and refinement

Crystals were cryo-protected with 15% glycerol added to the reservoir solution and flash-frozen with liquid nitrogen. A 2.7 Å resolution dataset was collected at 100 K using an in-house X-ray source (Rigaku MicroMax-007 desktop rotating-anode X-ray generator with a Cu target operated at 40 kV and 30 mA) and an R-AXIS VI++ imaging-plate detector with a 220 mm crystal-to-detector distance at a wavelength of 1.5418 Å. The crystal belongs to space group *P2*_*1*_ *2*_*1*_ *2*_*1*_ with unit cell dimensions *a* = 60.843 Å, *b* = 73.077 Å and *c* = 218.746 Å, α = β = γ = 90°. Diffraction data was processed, integrated and scaled with HKL2000 [37]. The structure of Alt_*Tt*_ was determined by molecular replacement using the alanine racemase from *Bacillus stearothermophilus* (Alr_*Bst*_, PDB ID 1SFT) as a search model and the PHASER program [38] from CCP4 package [39]. Iterative model building and refinement were performed using Coot [40] and Refmac5 [41] to obtain the final model with R_*work*_ of 21.3% and R_*free*_ of 25.2% at 2.7-Å resolution (Table 1).

**Table 1:** X-ray diffraction data collection and structure refinement statistics.

**Data Collection**	
Cell parameters (Å)	*a* = 60.843, *b* = 73.077, *c* = 218.746
	α = β = γ = 90°
Space group	*P2*_*1*_ *2*_*1*_ *2*_*1*_
Resolution (Å)	50 (2.75)– 2.70
No. of all reflections	108967
No. of unique reflections	26832
Completeness (%)	96.6 (83.4)
Redundacy	5.2 (3.4)
I/σI	10.1 (2.7)
R_merge_ † (%)	7.3 (26.1)
**Refinement**	
Resolution (Å)	109.4–2.7
Total No. of reflections	25425
No. of reflections used	24072
R_work_ / R_free_ (%)	21.3/25.2
No. of atoms	6237
Protein	3836
Water	0
ALA	2
PO4^3-^	2
R.m.s. deviations	
Bond lengths (Å)	0.007
Bond angle (°)	1.0
Average B-factors (Å^2^)	45.7

Values in parentheses are for the highest resolution shell.

^†^ *R*_merge_ = ∑_*hkl*_ ∑_*i*_ │*I*_*i*_ *(hkl)* -〈*I(hkl)*〉│/ ∑_*hkl*_ ∑_*i*_ *I*_*i*_ *(hkl)*, where *I*_*i*_ *(hkl)* is the intensity of the *i*th measurement of reflection *hkl* and〈*I(hkl)*〉is the mean intensity of all symmetry related reflection.

### Racemization enzyme assay

Micro-plate assay was carried out to measure the enzyme activity of Alr_*Tt*_ and its mutants. The racemization mixture was composed of PLP (10 μM), NaHCO_3_–NaOH buffer (50 mM, pH 10.0), L-Ala (50 mM) and appropriate amount of enzyme in a final volume of 200 μL. Buffer instead of enzyme was set as negative control. The reaction was carried out at 338 K for 10 min. D-forms amino acids were measured at 310 K with the coupling protein (D-amino acid oxidase and peroxidase) as described [17, 42]. The D-Amino acid oxidase reaction was performed using 200 mM Tris-HCl pH 8.0, 0.1 mg mL^-1^ 4-aminoantipyrine, 0.1 mg mL^-1^ TOOS, 2U peroxidase (Horseradish, Sigma) and 0.1U D-amino acid oxidase (Procine kidney, Sigma) at a volume of 200 μL. The absorption at 550 nm was recorded using Epoch Microplate Spectrophotometer (BioTek, USA). One unit (U) of the racemase activity was defined as the amount of enzyme consumed in formation of 1 μmol D- or L-Ala from either enantiomer per minute.

Kinetic parameters of alanine racemase Alr_*Tt*_ and its mutants were determined by measuring the amounts of D- and L-Ala by HPLC as previously described [43]. Protein concentration of Alr_*Tt*_ and its mutants was determined by BCA Protein Assay Reagent Kit (Pierce, USA) using bovine serum albumin (BSA) as a protein standard.

### Determination of the PLP content in the enzyme

PLP content of the enzyme was determined by a spectroscopic method [44]. Wild-type and mutant Alr_*Tt*_ were incubated with 10 mM hydroxylamine for 30 min at 310 K to remove PLP from active site [22], and then dialyzed with 10 mM Tris-Cl pH 8.0 to obtain the apo-enzyme. The absorption spectrum of the apo- and holo-enzyme of wild-type Alr_*Tt*_ were scanned from 250 to 500 nm using a spectrophotometer TU-1810 (Persee, China). The wavelength gives maximum absorption of the holo-enzyme was used for measuring the absorption of different concentrations of PLP (0, 2, 5, 10, 20, 50, 100, 200 μM), which was applied as the standard curve to determine the PLP content in the enzymes.

## Results and Discussion

### Overall architecture of Alr_*Tt*_ is similar to anabolic alanine racemase

**Fig 1:**
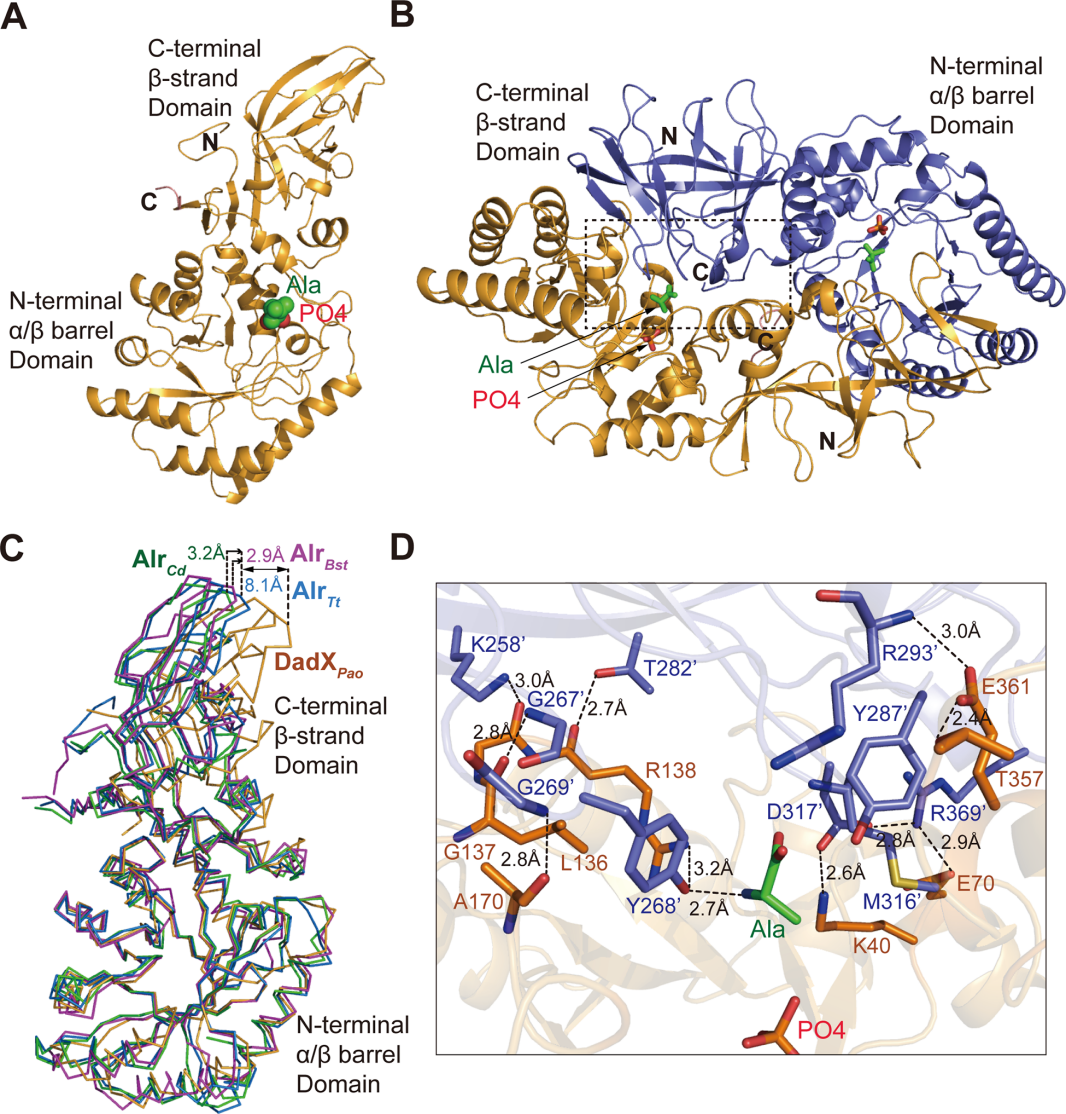
Overall crystal structure of Alr_*Tt*_. **(A)** Overall structure of Alr_*Tt*_ monomer. N-terminal α/β barrel domain, and the C terminal β-strand domain are shown in orange, phosphate group (red) and L-Ala (green) in the active site are shown in spheres. **(B)** Dimer of Alr_*Tt*_, it is formed by two head-to-tail associated monomers (colored in orange and blue) in one asymmetric unit. The dimer interface is indicated by dashed box. The phosphate group (red) and L-Ala (green) are shown in sticks. **(C)** Comparison of the overall architectures of alanine racemase from a gram positive bacteria *Bacillus stearothermophilus* (Alr_*Bst*_, PDB 1SFT, magenta), a gram negative bacteria *Pseudomonas aeruginosa* (DadX_*pao*_, PDB 1RCQ, orange), and *Clostridium difficile* strain 630 (Alr_*Cd*_, PDB 4LUS, green) with Alr_*Tt*_ (PDB 4Y2W, blue). The structures are superimposed at the N-terminal α/β barrel domain, the shift of the β-strand domain are represented by distance from Arg276 in Alr_*Tt*_ to corresponding residues like Arg261 in DadX_*pao*_, Thr273 in Alr_*Bst*_ and Gly276 in Alr_*Cd*_. **(D)** Hydrogen bonding interactions mediating the dimer formation, residues in N-terminal α/β barrel domain of one monomer (orange) and the C terminal β-strand domain of the another monomer (blue) are shown in sticks, the hydrogen bonds are indicated as dashed lines.

Preliminary crystallization and X-ray characterization of Alr_*Tt*_ is as described [36]. The structure is determined by molecular replacement method and refined to *R*_*work*_ of 21.3% and *R*_*free*_ of 25.2% at 2.7 Å resolution (Table 1). Like typical alanine reacemase, a homo-dimer of Alr_*Tt*_ was found in one asymmetric unit. Each monomer covers full-length Alr_*Tt*_ (Val1-Lys383), it contains an N-terminal eight-stranded α/β barrel domain (residues 1–244) and a C-terminal extended β-strand domain (residues 245–383) (Fig 1A). The active site is located in the center of α/β barrel domain, it is surrounded by parallel β strands (β2, β3, β4, β5, β6, β7, β8 and β9) in the inner layer, and α helices (h2, α3, α4, α5, α6, α7, α8, α9 and α10) at the outer layer (S1 Fig). One L-Ala molecule and a phosphate group were modeled in the refined structure (Fig 1A and 1B). Two identical monomers (with Cα atoms r.m.s. difference of 0.396 Å) associate at the C-terminal β-strand domain and the α/β barrel domain to form the functional dimer (Fig 1B).

Overall architecture of Alr_*Tt*_ is similar to bacterial alanine racemase, especially the N-terminal α/β barrel domain, it matches well with the same region of alanine racemase from a gram positive bacteria *Bacillus stearothermophilus* (Alr_*Bst*_, PDB 1SFT) [23], a gram negative bacteria *P*. *aeruginosa* (DadX_*pao*_, PDB 1RCQ) [31], and *Clostridium difficile* strain 630 (Alr_*Cd*_, PDB 4LUT) [29] (Fig 1C). Alr_*Tt*_ shares 41% amino acid sequence identity with Alr_*Cd*_, the r.m.s. difference of the Cα atoms between the two structures is 0.99 Å, whereas it is 1.245 Å for Alr_*Bst*_ and 2.145 Å for DadX_*Pao*_. Superimposing these alanine racemase structures at the N-terminal α/β barrel domain, the deviation occurs mostly at the β-strand domain of the catabolic alanine racemase DadX_*Pao*_, the tip region (Arg261) shifts about 8.1 Å away from Alr_*Tt*_ (Arg276), suggesting that the architecture of Alr_*Tt*_ is close to the anabolic alanine racemase Alr_*Bst*_ and Alr_*Cd*_ (Fig 1C), which is consistent with the observed enzyme activity of Alr_*Tt*_ and DadX_*Tt*_ [22].

### Two subunits of Alr_*Tt*_ associate as dimer to form the intact active site pocket

It has been reported that *E*.*coli* and *Pseudomonas aeruginosa* alanine racemase are active as dimer [45]. In addition, the racemase activity is correlated with the dimer formation. In some *Shigella* species, the monomeric alanine racemase forms dimer in catalytic reaction, when L-Ala or inhibitor D-cycloserine were added [20, 46]. However, size exclusion chromatography shows that both the wild-type and Q360Y Alr_*Tt*_ are monomers, even in presence of the substrate L-Ala and cofactor PLP (S2 Fig), the low enzyme activity of Alr_*Tt*_ probably makes it difficult to capture the dimer formation in solution.

Although Alr_*Tt*_ exists as a monomer in solution (S2 Fig), a homo-dimer was found in one asymmetric unit from the crystal structure. Most catalytic residues are associated at the dimer interface to form intact active site pocket, like Lys40, Tyr268’, Tyr287’, Arg293’, Asp317’ and Arg138 (Fig 1B and 1D). The key catalytic residue Tyr268’ is brought into the active site pocket through dimer formation, its hydroxyl group forms hydrogen bonds with L-Ala (2.7 Å) and side chain of Arg138 (3.2 Å) (Fig 1D). Another key catalytic base Lys40 forms hydrogen bond (2.6 Å) with carboxyl group of Asp317’, another carboxyl group of Asp317’ is hydrogen bonding with side chain of Arg369’ (2.8 Å), which is further stabilized by Glu70 (2.9 Å). Glu361 forms hydrogen bond with main chain nitrogen of Arg293’ (3.0 Å) and hydroxyl group of Thr357 (2.4 Å). Side chain of Arg293’ flips towards the active site pocket and lies in the middle layer of the substrate entryway. At the other side of the dimer interface, amino acids from loop regions of the β-strand domain (Gly267’-Gly269’) and the α/β barrel domain (Leu136-Arg138) closed the active site pocket through a clusters of hydrogen bonding interactions: Leu136-Gly267’ (2.8 Å), Gly137-Lys258’ (3.0 Å), Ala170-Gly269’ (2.8 Å). In addition, Thr282’ is constrained by interaction with main chain oxygen of Arg138 (2.7 Å). These hydrogen bonding interactions bring two subunits together to form a strictly constrained active site pocket, then the abstracted α-proton can be transferred between OH of Tyr268’, ε-amino group of Lys40 and the external aldimine to catalyze the alanine racemization. Thus, the homo-dimer conformation is necessary for forming the intact active site pocket.

### Presence of Gln360 and conformational changes of the active site residues result in improper PLP immobilization

Although we co-crystallized Alr_*Tt*_ with PLP and L-Ala, only L-Ala and the phosphate group of PLP were observed in the active site of Alr_*Tt*_, the pyridine ring is not interpreted due to lack of clear electron density. PLP could form an internal Schiff base with ε-amino group of Lys40 in the active site and show a high absorption at about 400 nm [22]. When wild-type Alr_*Tt*_ (0.81 mg mL^-1^) was scanned from 250 to 500 nm, a maximum absorption of PLP was observed at 405 nm. Then the absorption of different concentrations of PLP at 405 nm was plotted as standard curve to calculate the PLP content of wild-type and mutant Alr_*Tt*_. The PLP content of wild-type Alr_*Tt*_ was determined to be 1.44 ± 0.01 mol PLP per mol enzyme, which is lower than the mutant enzymes that mimic the conserved catalytic residues at the substrate entryway. The PLP content for the middle layer mutant S173D is 1.78 ± 0.02 mol PLP per mol enzyme, for the inner layer mutant Q360Y is 1.93 ± 0.02 mol PLP per mol enzyme, and the double mutant Q360YS173D is 1.96 ± 0.02 mol PLP per mol enzyme. PLP content in wild-type Alr_*Tt*_ is also lower than DadX_*Tt*_ (1.57 mol PLP per mol enzyme) [22] and DadXOF4 (2.16 mol PLP per mol enzyme) [17], suggesting a decreased PLP binding affinity of Alr_*Tt*_. Missing of the clear electron density of the pyridine ring may results from the improper immobilization of PLP in the active site pocket.

**Fig 2:**
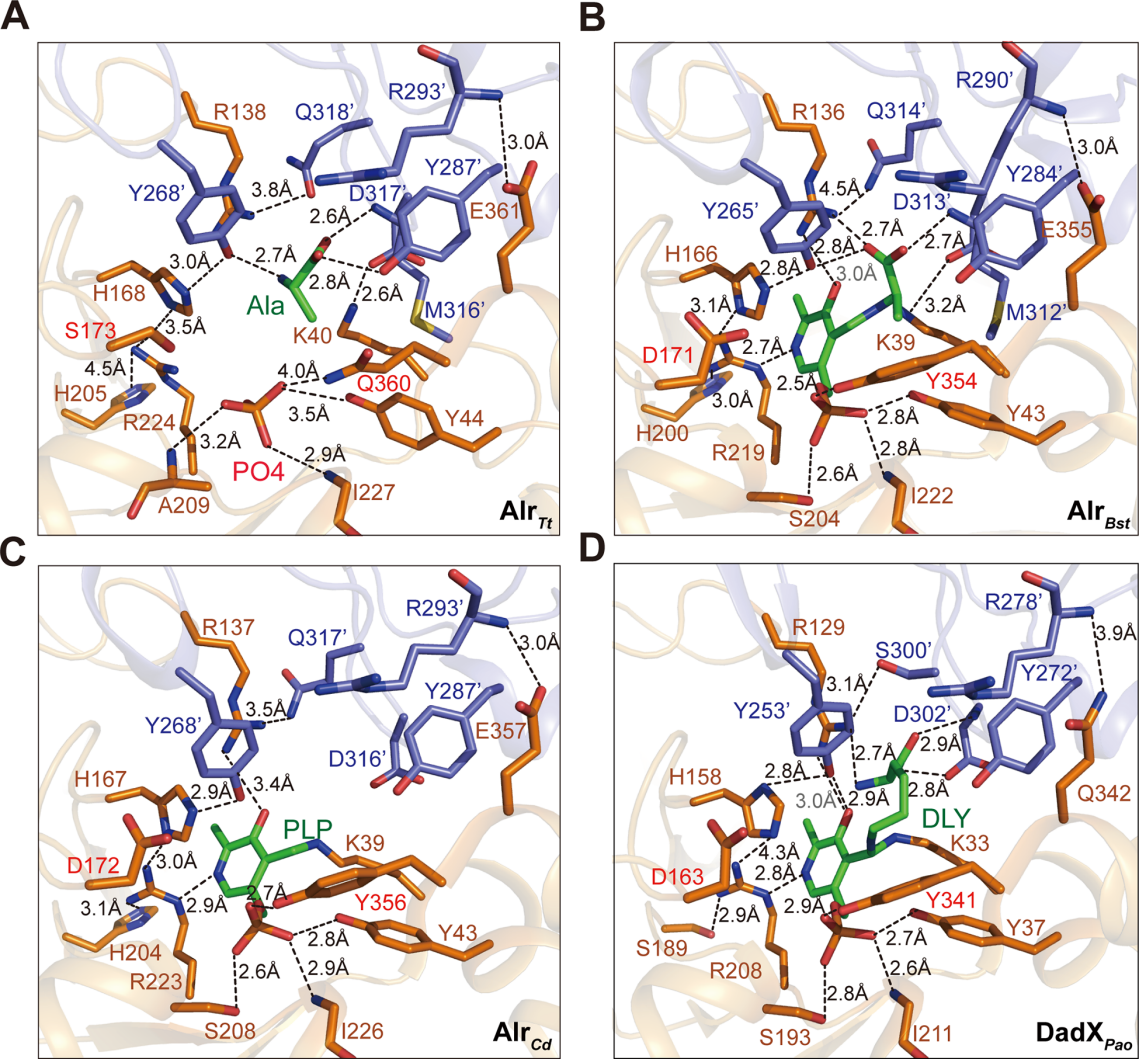
Comparison of the active site pocket of Alr_*Tt*_ with other three bacterial alanine racemases. **(A)** The active site pocket of Alr_*Tt*_ (PDB 4Y2W), phosphate group, L-Ala and key amino acids that involved in substrate binding are shown in sticks, the hydrogen bonding interactions are indicated. Same view of the active site pocket of other three bacteria alanine racemase in complex with substrates are shown in same profile: **(B)** the PLP-D-Ala complex of Alr_*Bst*_ (PDB 1L6G), **(C)** PLP complex of Alr_*Cd*_ (PDB 4LUS), **(D)** PLP and DLY complex of DadX_*pao*_ (PDB 1RCQ).

Dramatic conformational changes of the active site residues were observed in the structure, which could cause improper immobilization of the pyridine ring. First, side chain of the key catalytic base Lys40 forms a hydrogen bond with carboxyl group of Asp317’ (2.6 Å), disrupted the covalent bond with pyridine ring that is necessary for forming the internal aldimine (Fig 2A). Second, the hydrogen bonding interactions necessary for stabilizing the pyridine ring are also destroyed. In Alr_*Bst*_ PLP-D-Ala complex structure [30], the pyridine ring is positioned by hydrogen bonds with Arg219 at N1 atom (2.7 Å), and Arg136 at phenolic oxygen (O3’) (3.0 Å). The guanidinium moiety of Arg219 is part of a long hydrogen bonding network (His200-Arg219-His166’-Tyr265’, Fig 2B). Similar interactions are observed in PLP complex of Alr_*Cd*_ (His204-Arg223-His167-Tyr268’, Fig 2C) and the PLP-D-Lysine (DLY) complex of DadX_*pao*_ (Ser189-Arg208-His158-Tyr253’, Fig 2D). However, these interactions are disrupted or much weaker in Alr_*Tt*_: His205-Arg224 (4.5Å), Arg224-His168 (3.5Å), His168-Tyr268’ (3.0 Å) (Fig 2A). Thus, side chain of Arg224 is not properly coordinated by His205 and His168 to adopt an optimal orientation for stabilizing the N1 atom of the pyridine ring. As a result, the pyridine ring is improperly immobilized in the active site pocket, which yields weak electron density.

In addition, presence of Gln360 destabilized the hydrogen bonding interactions necessary for immobilizing the phosphate group of PLP. The free oxygen OP1 forms hydrogen bond with Ile227 (2.9 Å), OP3 forms weak hydrogen bond with Ala209 (3.2 Å). Compared to Tyr43 in Alr_*Bst*_, the aromatic ring of Tyr44 is rotated 90°, then the hydrogen bond with OP1 is weakened (3.5 Å). The short side chain of Gln360 disrupted the hydrogen bond with OP2 (4.0 Å), which is highly conserved in other bacterial alanine racemase (Fig 2A). In contrast, the phosphate group is strictly constrained by strong hydrogen bonding interactions in Alr_*Bst*_ PLP-D-Ala complex (Fig 2B): OP1 forms hydrogen bond with main chain nitrogen of Ile222 (2.8 Å) and hydroxyl group of Tyr43 (2.8 Å), OP2 forms hydrogen bond with OH of Tyr354 (2.5 Å), OP3 interacts with Ser204 (2.6 Å). Identical interactions are observed in DadX_*pao*_ (Fig 2C) and Alr_*Cd*_ (Fig 2D).

One L-Ala molecule is positioned in the active site pocket by hydrogen bonds with main chain nitrogen of Met316’ (2.6 Å), OH of inner layer residue Tyr287’ (2.8 Å) and Tyr268’ (2.7 Å). In Alr_*Bst*_ and DadX_*pao*,_ an additional hydrogen bond with Arg136 (2.7 Å) and Arg129 (2.7 Å) is observed (Fig 2B and 2D). The main chain nitrogen of Met316’ and the NH1 of Arg138 is responsible for constituting the recognition site for the carboxyl group of Ala [10, 23, 28]. However, due to missing of the pyridine ring, Arg138 is not contacting with either OXT of Ala or phenolic oxygen (O3’) of the pyridine ring like other alanine racemase (Fig 2A, 2C and 2D).

Based on the generally accepted two-base mechanism, Tyr268’ and Lys40 are the key catalytic bases that removes α-hydrogen from L- and D-Ala, and mediates the α-proton transfer in racemization reaction [30]. However, in Alr_*Tt*_, there is no internal aldimine observed, instead Lys40 forms hydrogen bond with Asp317’. Presence of Gln360 and conformational changes of Lys40, Arg224 and Tyr44 destroy the hydrogen bonding interactions necessary for stabilizing the pyridine ring and phosphate group, result in weaker PLP binding and low racemase activity of Alr_*Tt*_.

### Introduction of hydrophobic amino acids at Gln360 increased the racemase activity of Alr_*Tt*_

**Fig 3:**
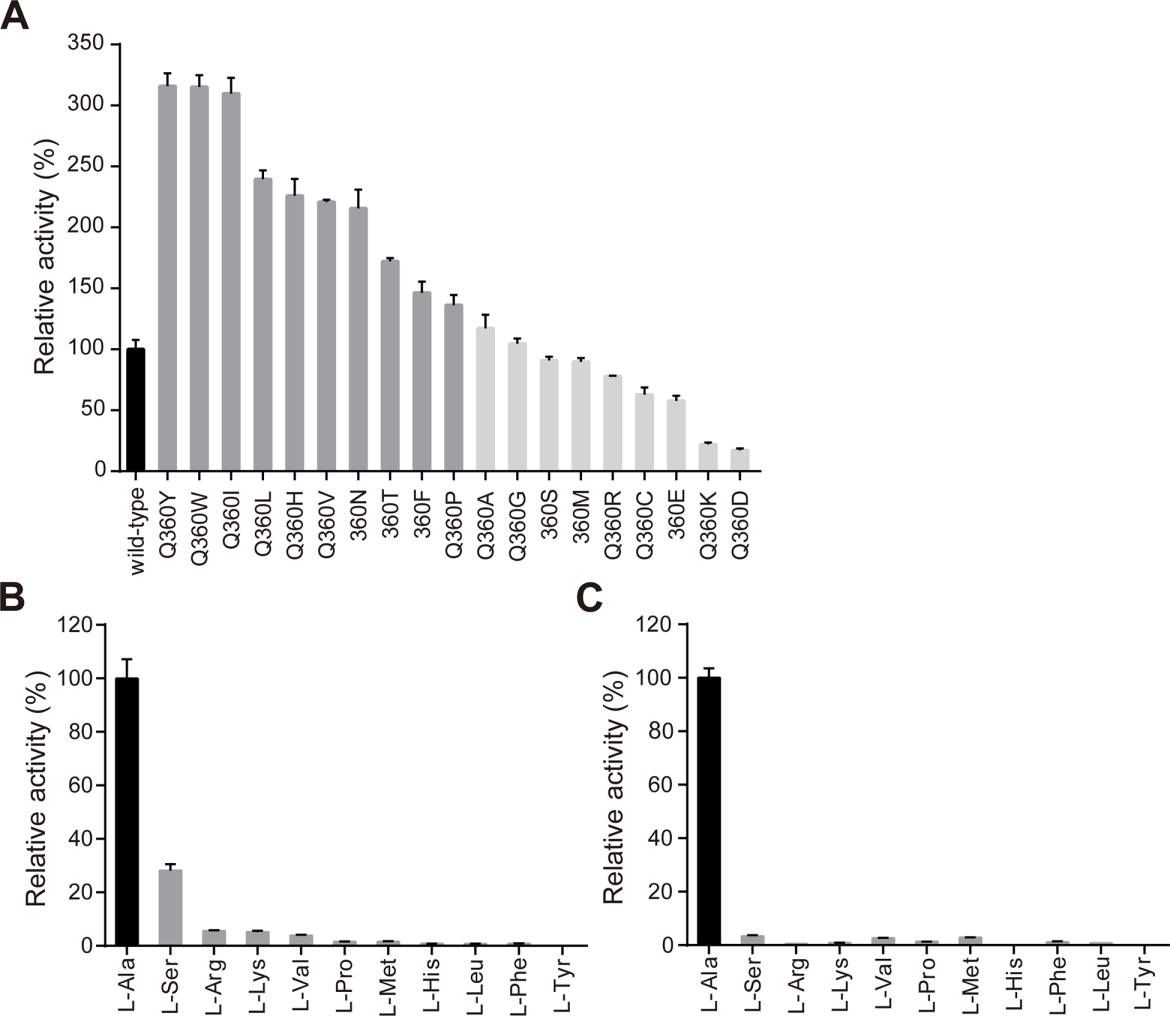
Relative activity of the saturation mutants of Gln360 in L-Ala racemization (A), and the relative activity of wild-type (B) and Q360Y mutant (C) in racemization of ten L-amino acids. **(A)** The racemase activities of the saturation mutants of Gln360 relative to wild-type Alr_*Tt*_ are represented by columns with means ± SD of quadruplicate experiments. **(B)** The racemase activities of ten L-amino acids relative to L-Ala catalyzed by wild-type Alr_*Tt*_ are represented by columns with means ± SD of quadruplicate experiments. **(C)** The relative amino acids specificity of Q360Y towards ten L-amino acids is shown in same profile as **(B)**.

To investigate the role of Gln360 in alanine racemization, we performed saturation mutagenesis of Gln360 and analyzed racemase activities of the mutants (Fig 3A). The racemase activity shows dramatic increase when Gln360 was mutated to hydrophobic amino acids, like aromatic residues (Phe, Tyr, Trp, His) and aliphatic residues (Ile, Leu, Val, Pro), enzyme activities of Q360T and Q360N also represented nearly 2 fold increase. When Gln360 was replaced by small amino acids like Ala, Gly and Ser, similar racemase activities to wild-type enzyme were observed. However, the activity decreased when Gln360 was mutated to charged amino acids like Arg, Lys, Asp and Glu. For amino acids carrying a sulphur atom, Q360M showed similar activity to wild-type, and Q360C represented 62.8% relative activity (Fig 3A and S2 Table).

In typical alanine racemase, Gln360 is highly conserved as a Tyr residue, it forms a hydrogen bond with phosphate group of PLP, which is believed to be essential for positioning the PLP cofactor in proper orientation for catalysis. However, there is no hydrogen bond between Gln360 and phosphate group in Alr_*Tt*_. In addition, mutation of Gln360 to hydrophobic and neutral amino acids that could not form hydrogen bonds surprisingly increased the racemase activity, indicating that forming hydrogen bond with PLP is not the primary role of residues at Gln360 position, instead its hydrophobic interaction with surrounding environment is more important in racemization.

**Fig 4:**
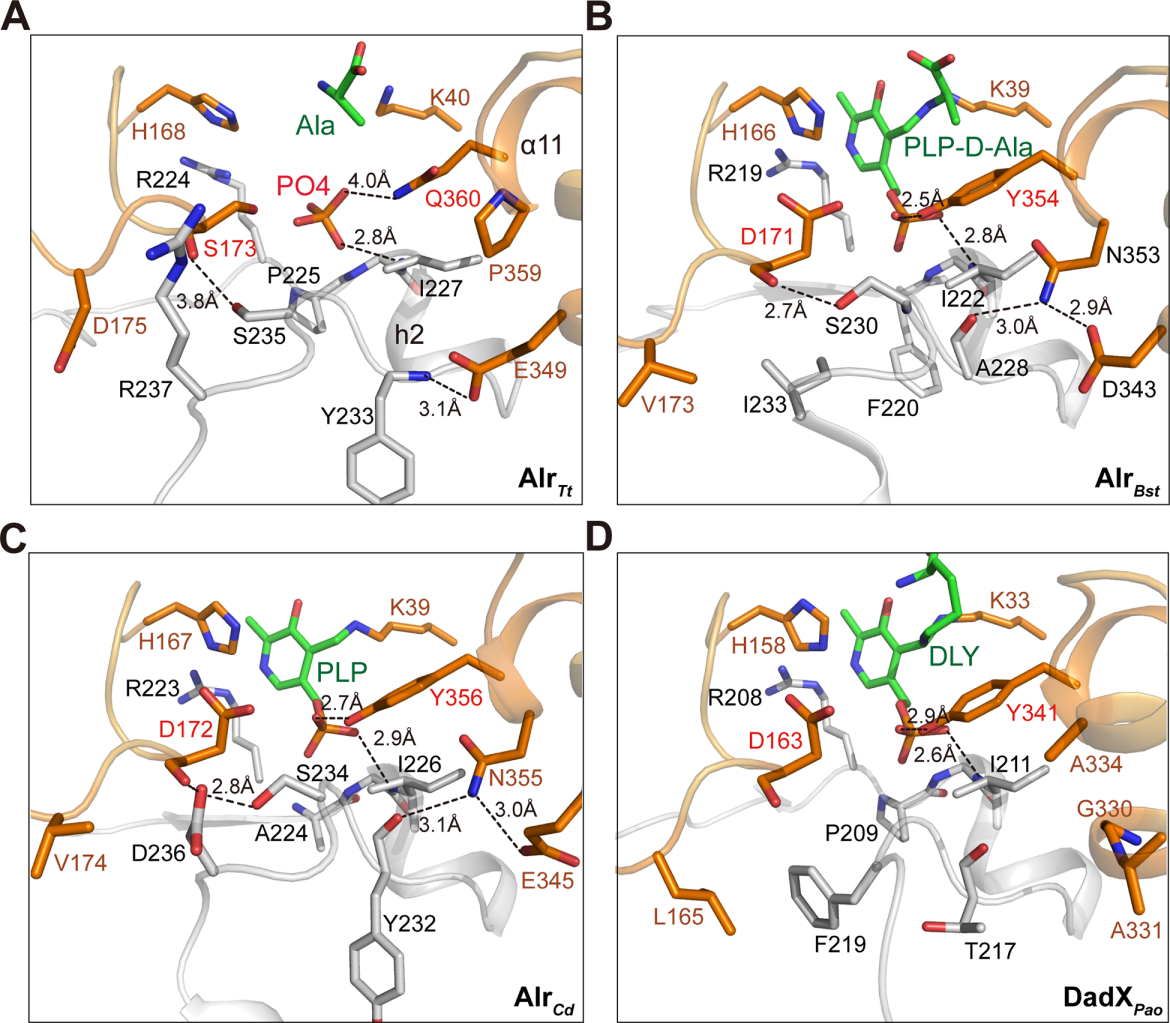
The hydrophobic patch and its interaction with active site pocket. **(A)** The hydrophobic patch (Pro225-Arg237) in Alr_*Tt*_ (PDB 4Y2W) is colored in gray, the active site pocket is shown in orange, the substrate and key amino acids mediating the interactions are shown in sticks, the hydrogen bonding interactions are indicated. The hydrophobic patch and active site pocket of the other three bacterial alanine racemases are shown in same view: **(B)** the PLP-D-Ala complex of Alr_*Bst*_ (PDB 1L6G). **(C)** PLP complex of Alr_*Cd*_ (PDB 4LUS), **(D)** PLP and DLY complex of DadX_*pao*_ (PDB 1RCQ).

**Fig 5:**
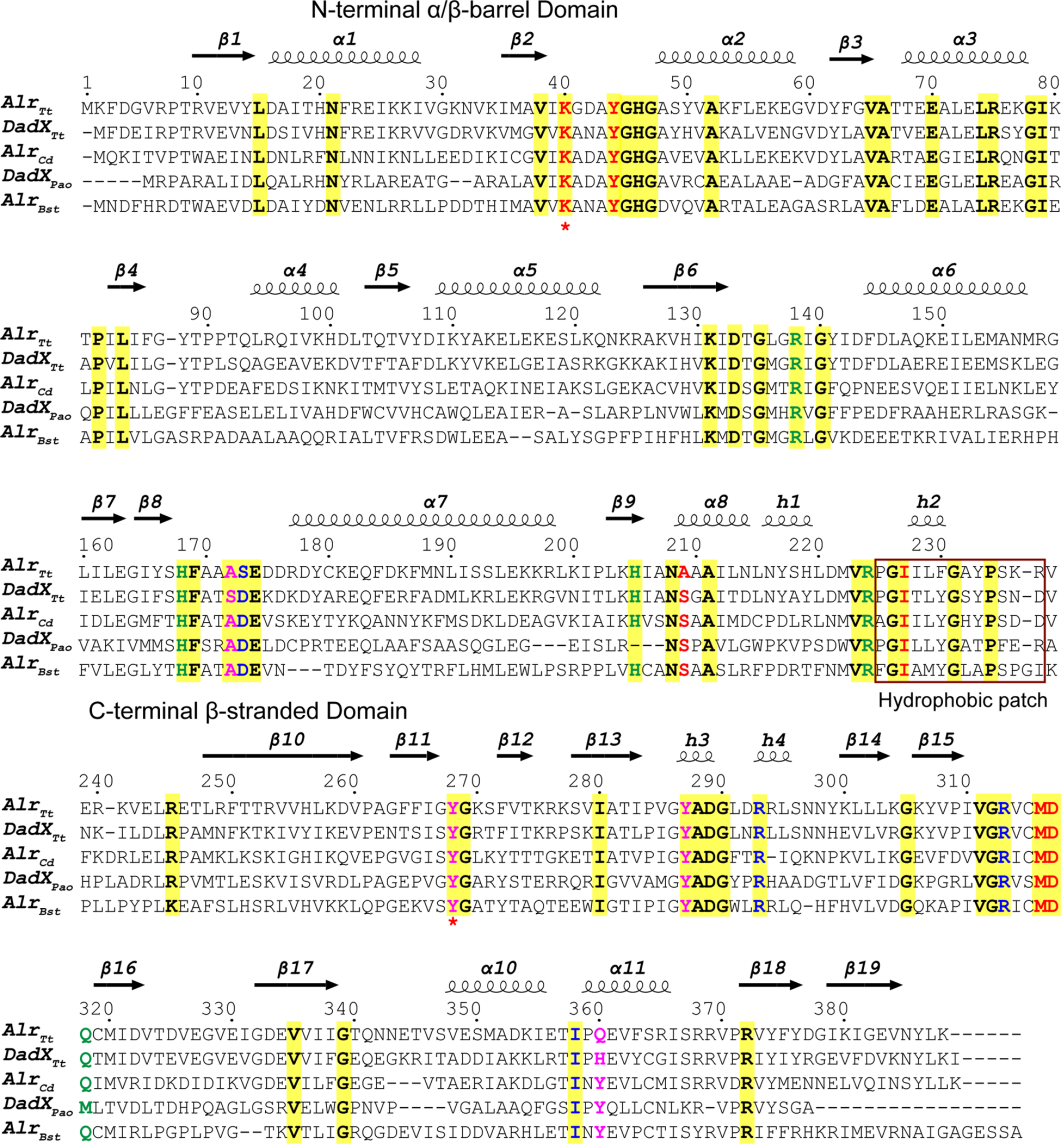
Structural based sequence alignment of Alr_*Tt*_, DadX_*Tt*_ and other three representative bacterial alanine racemases. Amino acid sequences of alanine racemase from a gram positive bacteria *Bacillus stearothermophilus* (Alr_*Bst*_), a gram negative bacteria *Pseudomonas aeruginosa* (DadX_*pao*_), and *Clostridium difficile* strain 630 (Alr_*Cd*_) are aligned with Alr_*Tt*_ and DadX_*Tt*_ from *T*. *tengcongensis* MB4. Amino acids are numbered and secondary structures are labeled, strictly conserved amino acids are highlighted in yellow box. Amino acids form the substrate entryway are colored in blue (middle layer) and magenta (inner layer), key catalytic residues mediating the phosphate group and L-Ala binding are colored in red, residues necessary for hydrogen bonding interactions for PLP-binding are colored in green. Two key catalytic residues Lys40 and Tyr268’ are marked with a star. The hydrophobic patch (Pro225-Arg337) in Alr_*Tt*_ is indicated by a red box.

In Alr_*Tt*_ structure, we observed a hydrophobic patch localized underneath Gln360 and phosphate group of PLP. This hydrophobic patch is conserved both in sequence and conformation among bacterial alanine racemase (Figs 4 and 5). It is formed by residues from Pro225 to Arg237 at helix h2 and Pro359 in α11. The backbone amide group of Gly226-Ile227 in this patch forms hydrogen bond with OP1 of the phosphate group (Fig 4A). In Alr_*Bst*_, A228 and Asp343 form hydrogen bonds with Asn353, dragging α11 into the active site pocket, thus side chain of Tyr354 is pointing towards the phosphate group to form the hydrogen bond (Fig 4B). Identical interactions are also observed in Alr_*Cd*_ (Fig 4C). In the catabolic alanine racemase DadX_*pao*_, the hydrophobic patch adopts similar conformation, but the hydrogen bonding are replaced by hydrophobic interactions. Although Ala334, Gly330 and Ala331 replaced the charged residues, α11 is still closely associated with the hydrophobic patch (Fig 4D).

When hydrophobic amino acids are introduced at Gln360, they first interact with Pro359 and residues like Ile227, Phe230 and Tyr234 in the hydrophobic patch to form a stable substrate binding pocket. Second, their side chains steric pack with the aromatic ring of Tyr44, which was given an optimal orientation for hydrogen bonding with the phosphate group. Thus, mutation of Gln360 to hydrophobic residues could enhance the hydrophobic interactions, provide a stronger steric hindrance to form a stable and strictly constrained substrate binding pocket for proper PLP immobilization, thus the racemase activity of Alr_*Tt*_ was increased. Smaller amino acids did not affect the racemase activity, indicating the side chain of Gln360 plays minor role in racemization of Alr_*Tt*_, which is consistent with the structural observations. However, when positive and negative charged residues were introduced, the long side chains and strong electric charges could disturb the hydrophobic interactions and positioning of the phosphate group, decreased the racemase activity.

### Hydrophobic amino acids at Gln360 enhanced both the substrate affinity and turnover of Alr_*Tt*_

To further understand the effects of these hydrophobic amino acids in alanine racemization, we analyzed the kinetic parameters of mutants Q360Y, Q360W and Q360I. Compared with wild-type, Q360Y mutation dramatically enhanced the overall catalytic efficiency (*k*_*cat*_ */K*_*m*_) of the enzyme, with the *K*_*m*_ value decreased about 10 fold for L-Ala and 7 fold for D-Ala, the turnover number (*k*_*cat*_) increased about 2 fold for L-Ala and 3 fold for D-Ala (Table 2). Mutation of Gln360 to Ile and Trp also enhanced the racemase activity with decreased *K*_*m*_ and increased *k*_*cat*_ value. However, Q360W mutation shows more effects on the turnover number than Q360I, it was increased 3 fold for L-Ala and 7 fold for D-Ala (Table 2).

**Table 2:** Kinetic parameters of wild-type and mutant Alr_*Tt*_.

	L-Ala→D-Ala			D-Ala→L-Ala			
Enzyme	*K*_*m*_ (mM)	*k*_*cat*_ (s^-1^)	*k*_*cat*_ */K*_*m*_	*K*_*m*_ (mM)	*k*_*cat*_ (s^-1^)	*k*_*cat*_ */K*_*m*_	*K*_*eq*_(L/D)
WT	780.20±25.89	212.38±4.09	0.27	381.22±9.27	96.86±10.71	0.25	1.07
Q360Y	87.81±8.72	431.46±22.52	4.91	52.03±5.41	251.21±18.82	4.83	1.02
Q360I	136.00±2.88	286.64±19.84	2.11	110.31±6.12	224.98±7.80	2.04	1.03
Q360W	176.11±13.22	612.43±30.63	3.48	216.09±4.28	633.45±45.52	2.93	1.18
S173D	157.53±10.42	34.69±1.57	0.22	160.92±13.72	32.54±0.94	0.2	1.09
Q360Y&S173D	83.10±8.78	246.48±8.11	2.97	58.48±7.29	133.08±7.17	2.27	1.31

In Alr_*Tt*_ structure, Ser173 is located at the loop connecting β8 and α7, it covers the active site pocket from top in middle layer of the substrate entryway (S1 Fig). Mutation of Ser173 to evolutionarily conserved Asp did not affect the overall catalytic efficiency of Alr_*Tt*_, it enhanced the substrate affinity but dramatically decreased the turnover number. Compared with Alr_*Bst*_ (Fig 4B) and Alr_*Cd*_ (Fig 4C), the loop region (His168-Asp175) shifts about 2 Å away the active site pocket, the hydrogen bond between main chain oxygen of Ser173 and Ser235 (3.8Å) is disrupted in Alr_*Tt*_ (Fig 4A). Mutation of Ser173 to Asp did not change this main chain mediated interaction, thus the overall catalytic efficiency was not affected.

Relative to S173D, Q360YS137D double mutation increased the turnover number 7 fold for L-Ala and 4 fold for D-Ala, and enhanced the substrate affinity for both L- and D-Ala. Additionally, Q360YS173D represented similar *K*_*m*_ value as Q360Y (Table 2), indicating that Gln360 plays more important role than Ser173 in racemization, it affects both the substrate affinity and turnover number of Alr_*Tt*_.

### Gln360 plays an important role in substrate selection of Alr_*Tt*_

We then analyzed the substrate specificity of Alr_*Tt*_ and Q360Y mutant towards other ten L-amino acids. Wild-type Alt_*Tt*_ shows low racemase activity towards hydrophobic and neutral amino acids, except that L-Ser represents 28% relative activity due to its similar architecture as L-Ala (Fig 3B and S3 Table). Q360Y mutant shows dramatically decreased racemase activity for L-Ser and other amino acids, indicating that introduction of Tyr at Gln360 enhances the substrate specificity of Alr_*Tt*_ (Fig 3C and S3 Table). Our observations are consistent with the report that Tyr354 in *Geobacillus stearothermophilus* alanine racemase is important for controlling the substrate specificity [33].

In the racemization reaction, Lys40 forms an internal aldimine with PLP, which will be replaced by external aldimine with the substrate L- or D-Ala. After Lys40 or Tyr268’ abstracts α proton from L- or D-Ala, the Ala side chain is rotated to form a planar carbanionic intermediate, it then points directly towards the side chain of Gln360. Introduction of hydrophobic residues at Gln360, especially those with bigger aromatic rings will yield stronger space hindrance to steric block side chain rotation of larger amino acids. So Q360W mutant contributes more effects on enhancing the turnover number of L- and D-Ala than Q360Y. In addition, these hydrophobic residues could form a gate at the substrate entrance with Tyr268’, which block entry of larger amino acids into the active site pocket. Thus, residues at Gln360 play an important role in substrate selection, mainly through hydrophobic interactions and steric block the larger amino acids entry and turnover. Our analysis provides an excellent explanation for the Tyr preference at Gln360 in other bacterial alanine racemase.

In DadX_*Tt*_, Gln360 is replaced by His359, this enzyme also shows much broader substrate specificity and higher catalytic efficiency than Alr_*Tt*_ [22]. This is consistent with our saturation mutagenesis studies, Q360H shows a 2.2 fold increase in the relative activity for L-Ala (Fig 3A and S2 Table). Interestingly, mutation of His359 (His359→Tyr359) in DadX_*Tt*_ dramatically decreases the enzyme activity [22]. Considering the catabolic properties and strong stability of DadX_*Tt*_, His359 may play additional roles in alanine racemization at extreme thermal environment.

## Conclusions

During evolution, most bacterial alanine racemase chose a Tyr residue at the inner layer of the substrate entryway to control substrate specificity. The thermostable *T*. *tengcongensis* MB4 contains two alanine racemase Alr_*Tt*_ and DadX_*Tt*_, both of them are not conserved at this position (Alr_*Tt*_: Gln360 and DadX_*Tt*_: His359). In this work, we determined the crystal structure of Alr_*Tt*_ in complex with L-Ala. Through comprehensive structural comparisons, saturation mutagenesis and enzyme activity analysis, we revealed the essential role of Gln360 in substrate selection of alanine racemization, and explained the preference of hydrophobic amino acids especially Tyr in other bacterial alanine racemase. Mutation of the conserved Tyr residue to Gln results in improper PLP immobilization, broadens the substrate specificity and decreases the racemase activity of Alr_*Tt*_. This observation inspires a new approach in antimicrobial drug development. An inhibitor could be designed to prevent the proper immobilization of PLP in alanine racemase, since PLP could still bind to the enzyme in this case, the non-specific binding of the inhibitor to eukaryotic PLP-dependent enzymes could be decreased. Considering the extreme growth conditions of *T*. *tengcongensis* MB4, the weaker PLP binding affinity might ensures a constitutively low racemase activity and broader substrate spectrum for D-Ala production, serving as an effective anti-stress response against the high temperature and pH environment.

## Supporting Information

S1 Fig Overview of the active site pocket of Alr_*Tt*_. Secondary structures involved in forming the active site pocket are indicated, phosphate group and L-Ala are shown in sticks. The non-conserved middle layer residue Ser173 and inner layer residue Gln360 at the entryway are shown in sticks and colored in red.
(TIF)

S2 Fig Size-exclusion chromatogram and SDS-PAGE analysis (insect) of wild-type and Q360Y mutant of Alr_*Tt*_. Purified Alr_*Tt*_ enzymes were loaded on a Superdex200 10/300 GL column (GE Healthcare) and eluted in buffer containing 25mM Tris–HCl pH 8.5, 200 mM NaCl and 10 uM PLP. Wild-type Alr_*Tt*_ at a concentration of 150 μM was incubated with 2.5 mM L-Ala and 300 μM PLP to mimic the racemization reaction. The chromatogram shows that both the wild-type and Q360Y mutant of Alr_*Tt*_ are monomers in solution.
(TIF)

S1 Table Primers used in the expression plasmid construction and saturation mutagenesis of Alr_*Tt*_. (DOC)

S2 Table The racemase activities of Gln360 saturation mutants compared to wild-type Alr_*Tt*_. (DOC)

S3 Table The racemase activities of ten L-amino acids relative to L-Ala catalyzed by wile-type and Q360Y mutant of Alr_*Tt*_. (DOC)
