# Supplementary figures and images for: Crystal Structure of a Thermostable Alanine Racemase from Thermoanaerobacter tengcongensis MB4 Reveals the Role of Gln360 in Substrate Selection
Source: PLoS One. 2015 Jul 28;10(7):e0133516. doi: 10.1371/journal.pone.0133516 (PMC4517790; doi:10.1371/journal.pone.0133516)

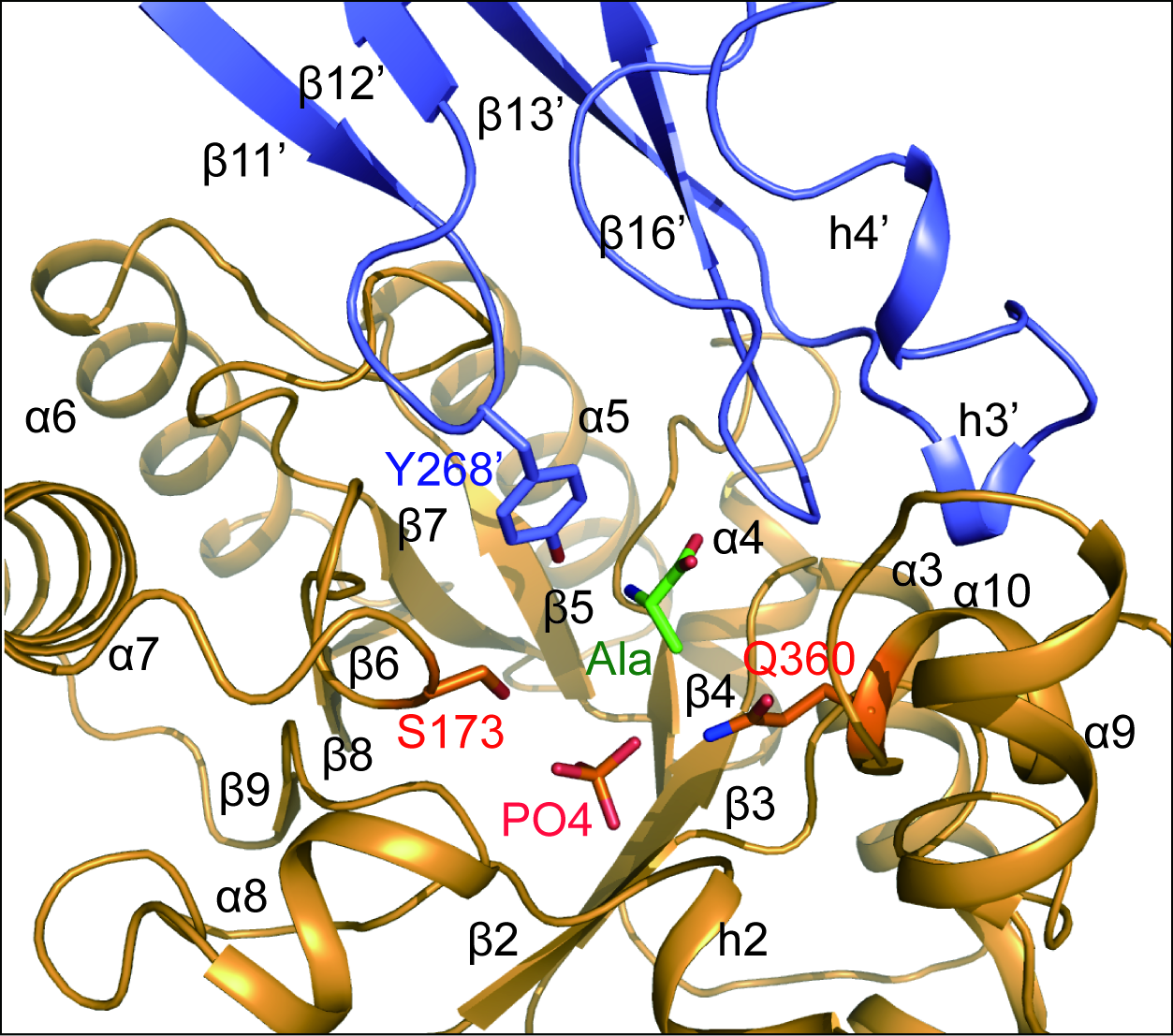

Supplement: S1 Fig — Secondary structures involved in forming the active site pocket are indicated, phosphate group and L-Ala are shown in sticks. The non-conserved middle layer residue Ser173 and inner layer residue Gln360 at the entryway are shown in sticks and colored in red. (TIF) [file pone.0133516.s001.tif]

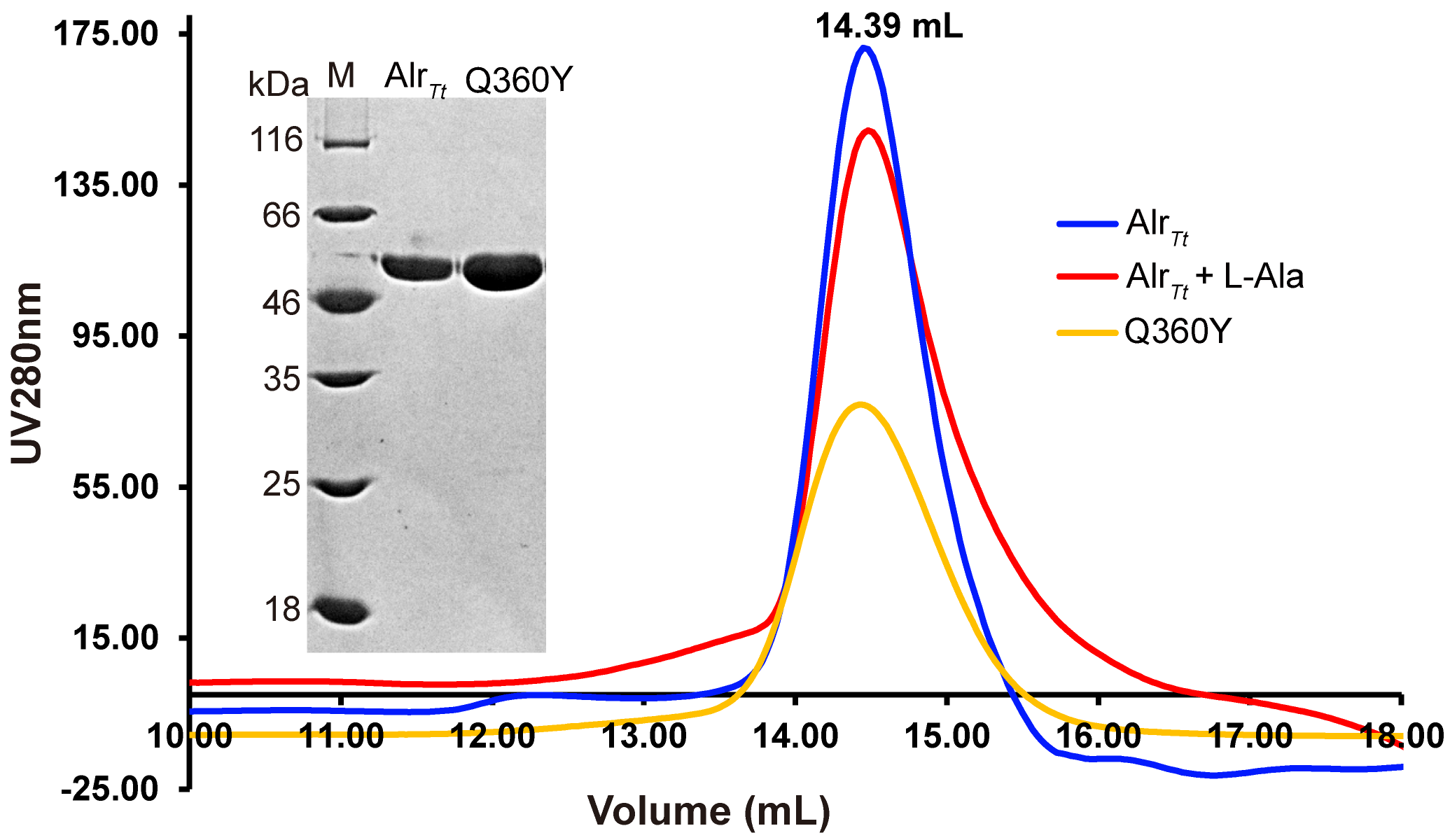

Supplement: S2 Fig — Purified AlrTt enzymes were loaded on a Superdex200 10/300 GL column (GE Healthcare) and eluted in buffer containing 25mM Tris–HCl pH 8.5, 200 mM NaCl and 10 uM PLP. Wild-type AlrTt at a concentration of 150 μM was incubated with 2.5 mM L-Ala and 300 μM PLP to mimic the racemization reaction. The chromatogram shows that both the wild-type and Q360Y mutant of AlrTt are monomers in solution. (TIF) [file pone.0133516.s002.tif]
