# Supplementary material for: Crystal Structure of a Thermostable Alanine Racemase from Thermoanaerobacter tengcongensis MB4 Reveals the Role of Gln360 in Substrate Selection
Source: PLoS One. 2015 Jul 28;10(7):e0133516. doi: 10.1371/journal.pone.0133516 (PMC4517790; doi:10.1371/journal.pone.0133516)
Supplement: S1 Table — (DOC) [file pone.0133516.s003.doc]

**S1 Table. Primers used in the expression plasmid construction and saturation mutagenesis of Alr*_Tt_*.**

| **Primer** | **Sequence (5′-3′)** | **Description** |
| --- | --- | --- |
| Alr*_Tt_* -F’ | GCTAGCGTGAAATTTGACGGGGTAAGA | *alr*_Tt_ cloning |
| Alr*_Tt_* -R’ | CTCGAGCTTTAAGTAATTTACTTCTCCA | *alr*_Tt_ cloning |
| S173D-F’ | TGCTGCCGCAgacGAAGATGAT | Ser173→Asp173 |
| S173D-R’ | ATCATCTTCgtcTGCGGCAGCA | Ser173→Asp173 |
| Q360Y-F’ | AACTATTCCTtatGAAGTTTTTTCT | Gln360→Tyr360 |
| Q360Y-R’ | AGAAAAAACTTCataAGGAATAGTT | Glr360→Tyr360 |
| S-Q360-F’ | GAAACTATTCCTnnsGAAGTTTTTTCT | Saturation mutagenesis |
| S-Q360-R’ | AGAAAAAACTTCsnnAGGAATAGTTTC |

The underlined sequences are *Nhe*I and *Xho*I recognition sites, respectively.
