# Supplementary material for: Crystal Structure of a Thermostable Alanine Racemase from Thermoanaerobacter tengcongensis MB4 Reveals the Role of Gln360 in Substrate Selection
Source: PLoS One. 2015 Jul 28;10(7):e0133516. doi: 10.1371/journal.pone.0133516 (PMC4517790; doi:10.1371/journal.pone.0133516)
Supplement: S2 Table — (DOC) [file pone.0133516.s004.doc]

**S2 Table. The relative racemase activities of Gln360 saturation mutants compared to wild-type Alr*_Tt_*.**

| **Enzyme** | **Relative Activity (100%)** |
| --- | --- |
| Wild-type | 100.00±7.77 |
| Q360W | 315.80±10.68 |
| Q360L | 315.28±9.71 |
| Q360I | 309.84±12.89 |
| Q360Y | 239.64±7.10 |
| Q360H | 226.05±13.69 |
| Q360V | 220.98±1.77 |
| Q360N | 215.63±15.38 |
| Q360T | 172.24±2.55 |
| Q360F | 146.36±9.31 |
| Q360P | 136.28±8.38 |
| Q360A | 117.21±11.27 |
| Q360G | 104.65±4.13 |
| Q360S | 90.84±3.09 |
| Q360M | 89.76±3.14 |
| Q360R | 77.98±0.52 |
| Q360C | 62.79± 5.90 |
| Q360E | 57.68± 4.36 |
| Q360K | 22.02±1.61 |
| Q360D | 17.21±1.46 |
