# Supplementary material for: Crystal Structure of a Thermostable Alanine Racemase from Thermoanaerobacter tengcongensis MB4 Reveals the Role of Gln360 in Substrate Selection
Source: PLoS One. 2015 Jul 28;10(7):e0133516. doi: 10.1371/journal.pone.0133516 (PMC4517790; doi:10.1371/journal.pone.0133516)
Supplement: S3 Table — (DOC) [file pone.0133516.s005.doc]

**S3 Table. The racemase activities of ten L-amino acids relative to L-Ala catalyzed by wile-type and Q360Y mutant of Alr*_Tt_*.**

| **L-amino acids** | **Wild-type** | **Q360Y** |
| --- | --- | --- |
| L-Ala | 100.00±7.19 | 100.00±3.56 |
| L-Ser | 28.13±2.45 | 3.35±0.45 |
| L-Arg | 5.60±0.28 | 0.54±0 |
| L-Lys | 5.13±0.57 | 0.89±0.052 |
| L-Val | 3.85±0.27 | 2.610±0.139 |
| L-Pro | 1.50±0.11 | 1.323±0.067 |
| L-Met | 1.58±0.16 | 2.830±0.130 |
| L-His | 0.82±0.04 | n.d. |
| L-Phe | 0.84±0.10 | 1.148±0.298 |
| L-Leu | 0.74±0.09 | 0.738±0.037 |
| L-Tyr | n.d. | n.d. |
